# Multivessel revascularisation versus infarct-related artery only revascularisation during the index primary PCI in STEMI patients with multivessel disease: a meta-analysis

**DOI:** 10.1007/s12471-015-0674-9

**Published:** 2015-03-13

**Authors:** S. Rasoul, V. van Ommen, J. Vainer, M. Ilhan, L. Veenstra, R. Erdem, L.A.W. Ruiters, R. Theunissen, J.C.A. Hoorntje

**Affiliations:** 1 Department of Cardiology, Maastricht University Medical Centre, PO Box 5800, 6202 AZ Maastricht, The Netherlands; 2 Atrium Medisch Centrum Heerlen, Heerlen, The Netherlands

**Keywords:** STEMI, Multivessel diseases, Multivessel PCI, Infarct-related artery

## Abstract

**Background:**

There are controversial data regarding infarct-related artery only (IRA-PCI) revascularisation versus multivessel revascularisation (MV-PCI) in ST-elevation myocardial infarction (STEMI) patients with multivessel disease undergoing primary percutaneous coronary intervention (PCI). We performed a meta-analysis comparing outcome in same stage MV-PCI versus IRA-PCI in STEMI patients with multivessel disease.

**Methods:**

Systematic searches of studies comparing MV-PCI with IRA-PCI in the MEDLINE and the Cochrane Database of systematic reviews were conducted. A meta-analysis was performed of all available studies. Primary outcome was all-cause mortality. Secondary endpoints were re-infarction, revascularisation, bleeding and major adverse cardiac events (MACE).

**Results:**

A total of 15 studies were identified with a total number of 35,975 patients. Mortality rate was significantly higher in the MV-PCI group compared with the IRA-PCI group, odds ratio (OR): 1.64 (1.46–1.85). Both the incidence of re-infarction and re-PCI were significantly lower in the MV-PCI group compared with the IRA-PCI group: OR 0.54 (0.34–0.88) and OR 0.67 (0.48–0.93), respectively. Bleeding complications occurred more often in the MV-PCI group as compared with the IRA-PCI group: OR 1.24 (1.08–1.42). Rates of MACE were comparable between the two groups.

**Conclusions:**

MV-PCI during the index of primary PCI in STEMI patients is associated with a higher mortality rate, a higher risk of bleeding complications, but lower risk of re-intervention and re-infarction and comparable rates of MACE.

## Background

About half of the patients presenting with ST-elevation myocardial infarction (STEMI) have multivessel disease. Compared with STEMI patients with single-vessel disease, STEMI patients with multivessel disease have a worse prognosis [1–3].

The current guidelines recommend intervention in the infarct-related artery only during primary percutaneous coronary intervention (PCI) except in haemodynamically unstable patients [4]; this is mainly due to the fact that evidence supporting immediate (preventive) intervention in the non-infarct-related artery is a matter of debate.

There are controversial data regarding infarct-related artery only revascularisation (IRA-PCI) versus multivessel revascularisation (MV-PCI) in STEMI patients with multivessel disease [5–19].

Previously, other meta-analyses assessed MV-PCI versus IRA-PCI; however, in those meta-analysis, MV-PCI was defined as same stage PCI as well as staged PCI days after the primary PCI. Furthermore, the results of the most recent trials were not included [20–23].

We performed a meta-analysis comparing outcome in MV-PCI versus IRA-PCI during the index of primary PCI in STEMI patients with multivessel disease.

## Methods

### Literature review

The literature search was performed from Cochrane Library, EMBASE and MEDLINE, from January 2014 to December 2014. The terms “ST-elevation myocardial infarction”, “coronary angioplasty”, “percutaneous coronary intervention”, “multi-vessel”, “non-culprit”, “culprit coronary revascularisation”, “complete revascularisation”, “myocardial infarction” and their variations were used as keywords. The search was limited to records in humans and English language articles.

### Study selection

Two reviewers independently screened all citations for eligibility. Both randomised controlled trials (RCTs) and cohort studies comparing multivessel versus culprit-only PCI in patients with STEMI and multivessel coronary artery disease treated with primary PCI were included. Studies enrolling patients with other than STEMI or comparing alternative revascularisation strategies were excluded. Full-text citations and abstracts were selected and independently screened for eligibility in the meta-analysis. The unpublished Complete Versus culprit-Lesion only PRimary PCI Trial (CVLPRIT) was also included because of its importance for this meta-analysis [20]. Quality of abstracted studies was assessed using the Cochrane Collaboration’s tool for assessing risk of bias [24].

Information on study design, inclusion and exclusion criteria, number of patients and clinical outcome was extracted by two investigators. Disagreements were resolved by consensus. Finally, all co-authors had full access to all study data and take responsibility for the integrity of the data and the accuracy of the data analysis.

### Definitions

MV-PCI was defined as PCI of the infarct-related artery (IRA) and non-IRA performed during the index primary PCI procedure for STEMI. IRA-PCI is defined as the PCI of the IRA only during the index primary PCI procedure. Major adverse cardiac event (MACE) was defined as the composite of death, re-infarction and revascularisation. Bleeding included both minor and major bleeding.

### Endpoints/data abstraction

The primary clinical endpoint was all-cause mortality. Secondary endpoints were re-infarction, revascularisation, bleeding and MACE.

### Statistical analysis

Continuous data were expressed as mean ± standard deviation and dichotomous data as absolute values and percentages. Mantel–Haenszel model was used to construct random effects summary odds ratios (ORs) and risk differences. All analyses were performed using Review Manager (RevMan, Version 5.0, The Nordic Cochrane Centre, The Cochrane Collaboration 2008) and SAS 9.3, (SAS Institute, Cary, NC). *p*-Value < 0.05 was considered statistically significant.

## Results

The search yielded 15 studies [5–19]: 5 RCTs and 10 cohort studies. The characteristics of the included studies are shown in Table 1. A total of 35,975 patients comprised the study population including 1134 (3.2 %) patients from RCTs. MV-PCI was performed in 5109 (12.2 %) patients, and 30,939 (85.8 %) patients underwent IRA-PCI.

**Table 1:** Study characteristics

Study	Design	Subjects	Inclusion criteria	Exclusion criteria	Primary endpoint	Mean length follow-up
Cavender	Cohort study	28,936	STEMI with CAD of > 1 major artery	LM, staged PCI (multiple PCIs before hospital discharge), thrombolytic	In-hospital mortality	In-hospital
Corpus	Cohort study	532	STEMI with > 70 % stenosis of ≥ 2 arteries	PCI of graft or after angioplasty, LM, planned staged revascularisation	MACE	12 months
Di Mario	Randomised	69	STEMI with MVD and 1–3 lesions in non-culprit artery technically amenable to revascularisation by stent	Lesion in vein and arterial grafts, prior angioplasty, thrombolytic, cardiogenic shock, LM	Repeat revascularisation	12 months
Dziewierz	Cohort study	777	STEMI with MVD 2–3 lesions in non-culprit artery	CABG	All-cause mortality	12 months
Hannan	Cohort study	1006	STEMI with MVD	LM disease, prior thrombolysis, prior CABG, cardiogenic shock, missing EF	All-cause mortality	42 months
Khattab	Cohort study	73	STEMI with > 70 % stenosis of ≥ 2 coronary arteries or major branches	Non-IRA diameter < 2.5 mm, LM disease, previous MI	MACE	12 months
Kornowski	Cohort study	668	STEMI with MVD	TIMI flow < 3 in non-IRA	MACE	12 months
Ochala	Randomised	92	STEMI with > 70 % stenosis of ≥ 2 coronary arteries, successful PCI of IRA	Cardiogenic shock, LM disease, pervious CABG, renal insufficiency, severe valvular disease	Improvement in LVEF	6 months
Politi	Randomised	214	STEMI with > 70 % stenosis of ≥ 2 coronary arteries or major branches	Cardiogenic shock, LM > 50 %, pervious CABG, severe valvular heart disease or unsuccessful procedure	MACE	30 months
Qarawani	Cohort study	120	STEMI with > 70 % multivessel narrowing	Cardiogenic shock, LM disease	Clinical outcome	12 months
Roe	Cohort study	129	STEMI with ≥ 50 % stenosis of ≥ 1 non-culprit artery in addition to culprit IRA	PCI of branch vessels of IRA, LM disease	MACE (death, re-MI, and revascularisation)	6 months
Toma	Cohort study	2201	STEMI with > 70 % stenosis of > 1 major epicardial artery and/or a non-IRA requiring intervention	PCI on LM, second intervention in the culprit artery	MACE (death, CHF, shock)	3 months
Varani	Cohort study	399	STEMI with > 70 % stenosis of ≥ 2 epicardial arteries or major branches	Occlusion after prior angioplasty, cardiogenic shock, pulmonary oedema	Death and repeat revascularisation	1 month
Wald	Randomised	465	STEMI with ≥ 50 % stenosis of ≥ 1 non-IRA in addition to IRA	Cardiogenic shock, LM > 50 %, pervious CABG	MACE	23 months
Gershlick	Randomised	294	STEMI with > 70 % stenosis of ≥ 2 epicardial arteries or major branches (> 2 mm)	Cardiogenic shock, previous MI, pervious CABG, chronic kidney disease, CTO	MACE	12 months

*CABG* coronary artery bypass graft, *CAD* coronary artery disease, *CHF* congestive heart failure, *CTO* chronic total occlusion, *IRA* infarct-related artery, *LM* left main artery, *LVEF* left ventricular ejection fraction, *MACE* major adverse cardiac events, *MI* myocardial infarction, *MVD* multivessel disease, *PCI* percutaneous coronary intervention, *STEMI* ST-elevation myocardial infarction, *TIMI* thrombolysis in myocardial infarction

### Patient characteristics

Table 2 shows the baseline characteristics of the study population. The vast majority of the studies excluded patients with cardiogenic shock and in two trials cardiogenic shock was not reported.

**Table 2:** Baseline characteristics

	Age		Male (%)		Diabetes (%)		Anterior MI (%)		Cardiogenic shock (%)	
Study	MV-PCI	IRA-PCI	MV-PCI	IRA-PCI	MV-PCI	IRA-PCI	MV-PCI	IRA-PCI	MV-PCI	IRA-PCI
Cavender	60	62	71.5	72.1	24.7	23.4	NR	NR	13.8	10.3
Corpus	64	63	70	70	19	17	NR	NR	3.3	3.4
Di Mario	64	65	88.2	84.6	11.5	41.5	51.9	58.8	Excluded	Excluded
Dziewier	68	68	72.2	72.2	NR	NR	NR	NR	Not reported	Not reported
Hannan	NR	NR	77.5	75.5	23.7	21.4	NR	NR	Excluded	Excluded
Khattab	69	65	75	78	7	16	57	54	3.6	4.4
Kornowski	62	63.5	80.9	79.6	15.3	18.1	40.6	35.1	Not reported	Not reported
Ochala	65	67	72.9	75	31	34	45.8	45.4	Excluded	Excluded
Politi	65	65	76.9	77.8	14	21	48	43	Excluded	Excluded
Qarawani	66	67	62	61	13	16	51	52	Excluded	Excluded
Roe	64	63	77.2	65.8	37	29	46	41	28	28
Toma	64	64	74	73	12	20	56	48	3	3
Varani	69	67	68.7	67	NR	NR	49	34	Excluded	Excluded
Wald	62	62	76	81	35	48	29	39	Excluded	Excluded
Gershlick	65	65	85	77	12.9	14.3	36	35.6	Excluded	Excluded

*IRA-PCI* infarct-related artery only revascularisation, *MI* myocardial infarction, *MV-PCI* multivessel revascularisation, *NR* not reported

### Clinical outcomes

**Fig. 1:**
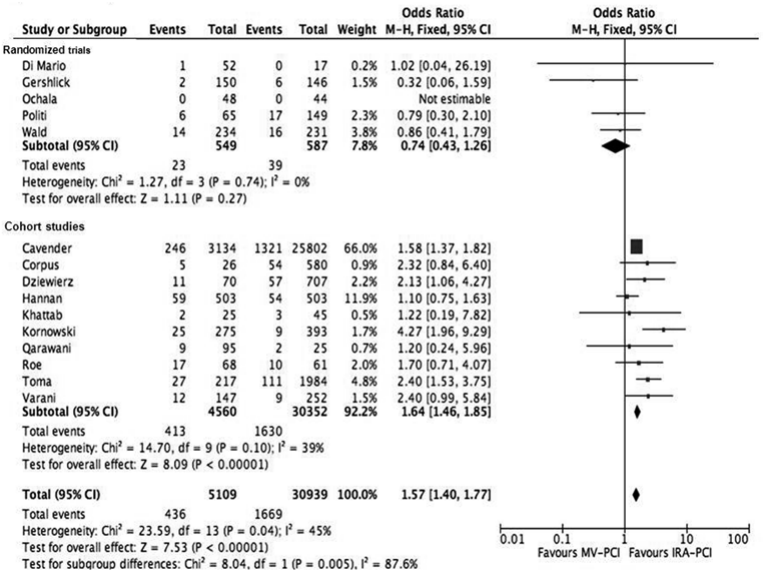
Forest plot of all-cause mortality

The primary endpoint, all-cause mortality, was significantly higher in the MV-PCI (8.5 %) compared with the IRA-PCI (5.4 %) group (OR 1.57, 95 % CI 1.40–1.76, *p* < 0.001) (Fig. 1). However, analysis limited to the five RCTs only showed no significant difference in mortality rate between MV-PCI and IRA-PCI (OR 0.74, 95 % CI 0.43–1.26, *p* = 0.27).

### Secondary endpoints

**Fig. 2:**
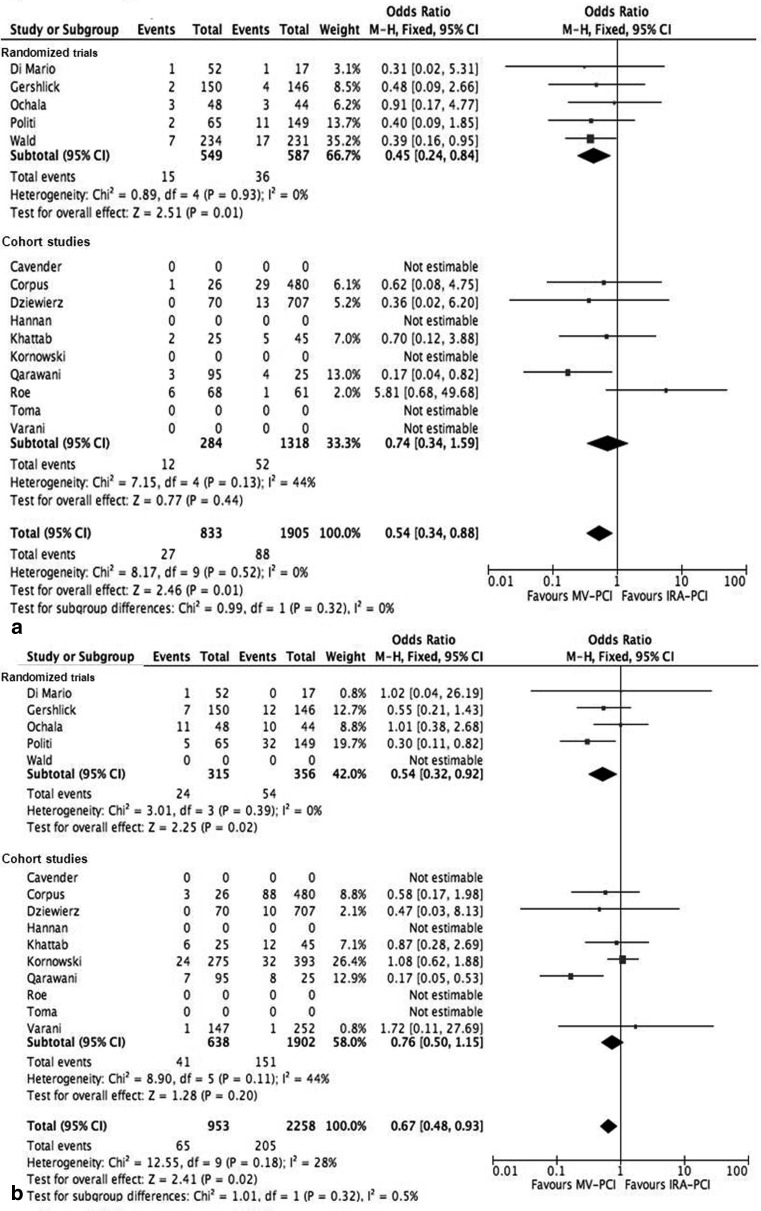
**a** Forest plot of re-infarction. **b** Forest plot of re-percutaneous coronary intervention

Rates of re-infarction (OR 0.54, 95 % CI 0.34–0.88, *p* = 0.01) and revascularisation (OR 0.67, 95 % CI 0.48–0.93, *p* = 0.002) were lower in the MV-PCI group. This was found for both randomised and cohort trials (Fig. 2a and b).

**Fig. 3:**
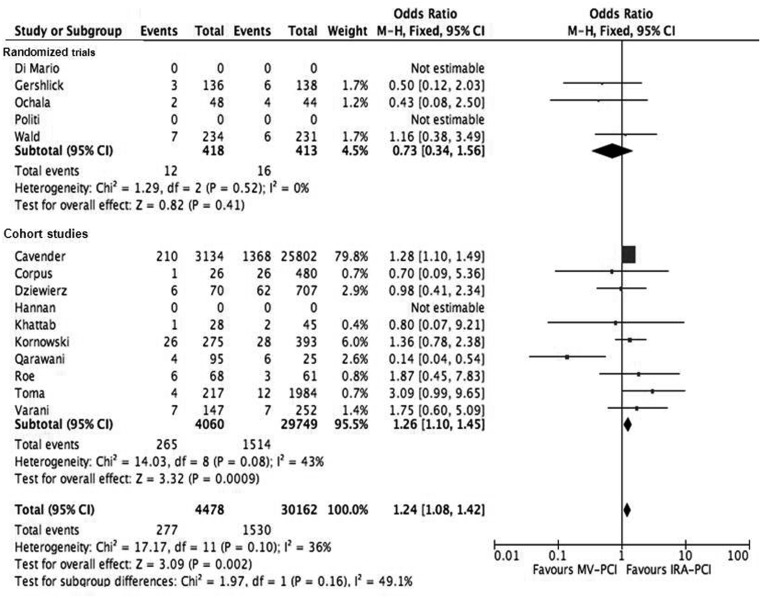
Forest plot of bleeding (major and minor)

Bleeding complications (major and minor) occurred more often in the MV-PCI group: 6.2 versus 5.1 %, (OR 1.24, 95 % CI 1.08–1.42, *p* = 0.002) and this was mainly found in the cohort studies (Fig. 3).

**Fig. 4:**
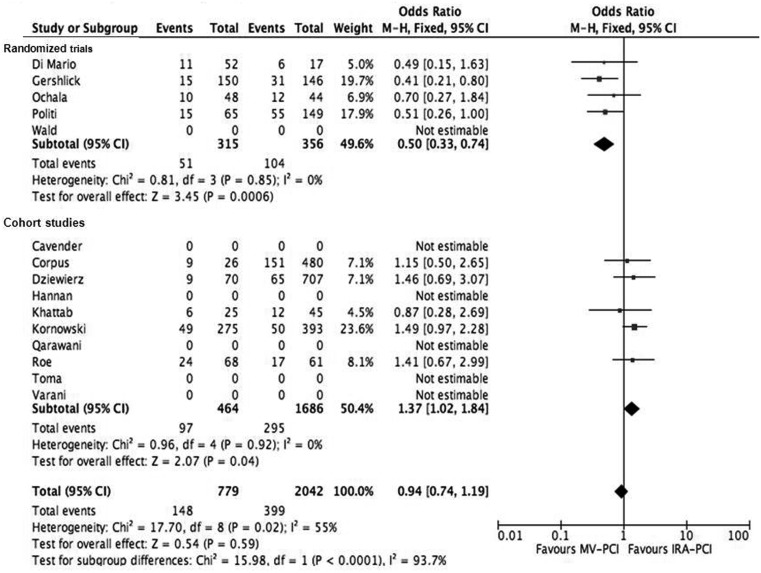
Forest plot of major adverse cardiac events (death, re-infarction and re-percutaneous coronary intervention)

MACE was comparable between the two groups: 19 versus 19.5 % (OR 0.94, 95 % CI 0.74–1.19, *p* = 0.59). In the RCT trials, MACE was significantly lower in patients undergoing MV-PCI compared with the IRA-PCI group (Fig. 4).

## Discussion

In this large scale meta-analysis, we found that PCI of the IRA and non-IRA performed during the index primary PCI procedure for STEMI, compared with IRA-only PCI, is associated with a higher mortality rate and more bleeding complications, but less re-infarction and revascularisation. Rates of MACE were comparable between the two groups. However, there was a clear difference in outcome between the randomised trials and cohort studies. In the cohort studies, mortality and bleeding complications were significantly higher in the MV-PCI group; however, these were not significantly different in the randomised trials between the MV-PCI group versus IRA-PCI group (Figs. 1 and 4).

Approximately 40–65 % of patients with STEMI have multivessel disease with increased risk of morbidity and mortality compared with single-vessel disease [1–3]. The underlying mechanism for this adverse prognosis may be plaque instability, impaired myocardial perfusion and contractility, arrhythmia and death.

The potential advantages of MV-PCI during the index primary PCI may prevent recurrent ischaemia and infarction by decreasing total ischaemia and improvement in myocardial function [25, 26]. Plaque instability may not be limited to the IRA but may involve other territories in the coronary vasculature. Moreover, complete revascularisation has been associated with improved long-term clinical outcome in patients with stable coronary artery disease. Finally, patients and clinicians may be more comfortable with complete revascularisation rather than medical therapy for angiographically significant residual coronary stenosis, especially if they are associated with a large territory of myocardial jeopardy [27–30].

However, multivessel PCI also has disadvantages. In the acute phase of STEMI, intervention of a non-culprit lesion may result in unnecessary haemodynamic compromise during PCI with balloon inflations or vessel-related complications (dissection, no-reflow) at a time when the patient has regional myocardial compromise. Given the extended duration of the intervention, increased contrast load and additional adverse peri-procedural outcomes may occur. Another important concern is poor assessment of lesion severity in non-culprit artery [22]. Hanratty et al. [30] demonstrated that 21 % of the non-culprit lesions are overestimated at time of AMI, and this may affect unnecessary revascularisation and inappropriate decision making. The severity of the non-culprit artery was judged visually and PCI of the non-IRA was not ischaemia guided in any of the studies included in this meta-analysis.

There is only one randomised study in which revascularisations on the non-IRA was guided by fractional flow reserve (FFR). FFR of the non-IRA was performed 7.5 days after primary PCI, and they found functional stenosis severity of non-culprit lesions is frequently overestimated and invasive strategy for non-culprit lesions did not lead to an increase in ejection fraction or a reduction in MACE [31].

Prior meta-analyses in this area have reported varying results due to differences in study design, comparison of different groups and different analytical methods [20–23]. Vlaar et al. [20] found that the strategy of staged PCI resulted in lower short- and long-term mortality compared with MV-PCI or IRA-PCI. Bangalore et al. [21] found that MV-PCI compared with IRA-PCI resulted in similar long-term mortality but a lower long-term rate of MACE. A recent meta-analysis showed that MV-PCI compared with IRA-PCI resulted in worse outcomes in cohort studies, but not in the randomised clinical trials [22]. This is in line with our findings.

Furthermore, Bainey et al. [23] found that staged multivessel PCI was superior to multivessel PCI during the index procedure.

The difference in outcome between the IRA-only and MV-PCI group may not only be due to revascularisation, differences in baseline may also play an important role. Patients in the MV-PCI group have a higher baseline risk evidenced by a higher proportion of anterior myocardial infarction and more cardiogenic shock.

Based on the current evidence, we think that in the acute phase of STEMI, revascularisation should be limited to the IRA only, except in patients with haemodynamic instability, as recommended by the current guidelines [4]. Staged and ischaemia-driven revascularisation of non-culprit lesions may be the treatment strategy for STEMI patients with multivessel disease. Further studies are needed to confirm this. The current ongoing COMPLETE and COMPARE ACUTE trials are studying these issues.

## Limitations

This meta-analysis was not performed on individual patient data. Caution should be exercised in the interpretation of the results, given the potential clinical heterogeneity among trials, due to varying patient populations and potential treatment bias. No information was available with regard to extent of coronary disease, use of drug-eluting stents, duration of dual antiplatelet therapy and access site. The short follow-up period of some studies is another important limitation. Furthermore, only a minority of the patients (14.2 %) undergo MV-PCI during the index procedure, so it is hard to draw definitive conclusions based on this meta-analysis.

In addition, no information was available regarding referral method, ambulance versus referring via non-PCI centres, factors that may affect total ischaemic time [32].

Finally, although the STEMI and non-STEMI are not uniquely related to different pathophysiological mechanisms [33], our results cannot be applied to non-STEMI patients with multivessel disease.

## Conclusion

Multivessel PCI during the index of primary PCI in STEMI patients is associated with a higher mortality and more bleeding, but a lower risk of re-intervention and re-infarction. Additional large-scale randomised trials are needed to guide the therapy and the timing for these patient subsets.

### Funding

None.

### Conflict of interest

None.
